# Automated Street Light Adjustment System on Campus with AI-Assisted Data Analytics

**DOI:** 10.3390/s23041853

**Published:** 2023-02-07

**Authors:** Somrudee Deepaisarn, Paphana Yiwsiw, Sirada Chaisawat, Thanakit Lerttomolsakul, Leeyakorn Cheewakriengkrai, Chanon Tantiwattanapaibul, Suphachok Buaruk, Virach Sornlertlamvanich

**Affiliations:** 1 School of Information, Computer, and Communication Technology, Sirindhorn International Institute of Technology, Thammasat University, Pathum Thani 12120, Thailand; 2 Faculty of Engineering, Thammasat University, Pathum Thani 12120, Thailand; 3 Faculty of Data Science, Asia AI Institute, Musashino University, Tokyo 135-8181, Japan

**Keywords:** smart city, smart street lighting, Internet of Things (IoT), artificial intelligence, web application, automated system, data analytics, environmental sensors

## Abstract

The smart city concept has been popularized in the urbanization of major metropolitan areas through the implementation of intelligent systems and technology to serve the increasing human population. This work developed an automatic light adjustment system at Thammasat University, Rangsit Campus, Thailand, with a primary objective of optimizing energy efficiency, while providing sufficient illumination for the campus. The development consists of two sections: the device control and the prediction model. The device control functionalities were developed with the user interface to enable control of the smart street light devices and the application programming interface (API) to send the light-adjusting command. The prediction model was created using an AI-assisted data analytic platform to obtain the predicted illuminance values so as to, subsequently, suggest light-dimming values according to the current environment. Four machine-learning models were performed on a nine-month environmental dataset to acquire predictions. The result demonstrated that the three-day window size setting with the XGBoost model yielded the best performance, attaining the correlation coefficient value of 0.922, showing a linear relationship between actual and predicted illuminance values using the test dataset. The prediction retrieval API was established and connected to the device control API, which later created an automated system that operated at a 20-min interval. This allowed real-time feedback to automatically adjust the smart street lighting devices through the purpose-designed data analytics features.

## 1. Introduction

One of the ten key messages from the United Nations predicts that the world population is expected to reach 8 billion in 2022 and to reach its peak at over 10 billion by the 2080s [1]. Traditional infrastructures in cities could not reach global standards everywhere in all aspects according to the current rate of population increase, along with the rapid change in global civilization, which has led to both development and disruption in many major cities around the world. The concept of a smart city was introduced and became widely popular, based on the development of various smart devices, applications of the Internet of Things (IoT), and global attention on improvement in the quality of life [2]. The six characteristics that described the smart city concept, according to Giffinger et al. [3] and Perera et al. [4], are smart governance, smart living, smart economy, smart people, smart mobility, and smart environment. Throughout this paper, the focus is on smart environment characteristics in the smart city motivating the implementation of an automatically adjusted smart street lighting system with the application of artificial intelligence enabling the light prediction functionality.

One of the examples is the implementation at Eindhoven, in the Netherlands, where the first set of smart street lighting implementations was introduced to the public in 2014, with the expectation of reducing carbon dioxide (CO_2_) emissions [5]. This also included a computer system to control each lighting device individually and automatically based on the circumstances in the area, such as weather and pedestrian conditions. Nowadays, there are various approaches and initiatives in different cities that build up smart city concepts to provide their citizens with a more convenient and improved quality of life through the renovation of their current existing infrastructure and digital transformation at all levels. Other adaptations of smart street light systems can be seen in Amsterdam, the Netherlands, under the Amsterdam Smart City (ASC) project, which is equipped with remotely controllable light devices adjusted in accordance with the surrounding environment, with similar focuses on energy and carbon dioxide emissions [6]. Barcelona, Spain also has a solution for the smart street light system that is equipped with a light-emitting diodes (LED) system for more energy efficiency [7] and an established application programming interface (API) to exchange information between smart lighting device management systems and other management systems, such as traffic management [8]. This extends their system toward a more intelligent system through the data collection of various sensors and expansion of knowledge of the city [9]. Similar implementations can also be seen in Chicago, Illinois, the United States, with their replacement of obsolete high pressure sodium (HPS) lights with LED lights and a new management system to promote energy savings of up to approximately 10 million US Dollars each year and to improve the overall safety of the city [10]. An example of such a system located geographically near Thailand can be seen in Singapore’s approach to the smart street lighting system. The Land Transport Authority of Singapore implemented LED street lighting, which is expected to reliably improve energy efficiency by 25 percent and reduce maintenance costs [11]. Their notable implementation is the introduction of a remote control and monitoring system (RCMS) that is responsive to variations in weather conditions [12].

For an application of AI modeling, Pizzuti et al. report that adaptive street lighting control, based on predicted traffic conditions, could save energy dramatically with an upper bound of 50% compared to the system without adaptive functions [13]. This finding is not applicable to large-scale cities, as the adaptive functions rely heavily on a thorough understanding of traffic conditions. While some studies focus on the adjustment of lighting levels according to traffic or density of population in the area, many also consider weather-related conditions with a view to understanding the context of the city to make policies that satisfy both lighting requirements and energy-saving aspects. To the best of our knowledge, available street lighting systems collect environmental data for the sake of gaining inferences. No such implementation of a street lighting system has proposed an AI-based lighting adjustment model using weather-related parameters. Hence, the novelty of our work is on building and optimizing an AI analytics model to provide a solution for automated street lighting adjustment in a campus area via illuminance prediction.

Moreover, several scientific articles in the weather-forecasting field indicate the lack of AI applications to predict radiation parameters, e.g., illuminance [14], which is also highlighted in Oh et al. [15]. Conventional machine learning and deep learning techniques are popular for solving numerical weather prediction problems [14] due to their ability to deal with non-linearity calculation [16]. However, deep learning approaches generally require extensive amounts of data for training to achieve optimal model performance. Less complicated machine learning models are also preferable, since the state-of-the-art models are likely to introduce drastic overfitting [17]. In 2021, Hanoon et al. applied conventional machine learning to predict weather values, i.e., temperature and relative humidity, attaining a correlation coefficient of predicted and actual values in the order of 0.7 and 0.6, respectively, using a model trained with up to six days of window size [16]. The result showed that varying the number of days of training data did not significantly affect the performance of machine learning models. These modeling techniques perform well on small offset predictions as they carry fewer cumulative errors. This motivated our attempt to predict illuminance values for the next day using simple machine learning models trained with data from a few days earlier. Our model was expected to obtain more or less similar performance to the work of Hanoon and co-workers, due to the similar characteristics of the weather data, experimental pipeline, and method of validation, except that the predicting parameters were different. Their temperature and our illuminance parameters are physical properties that are dependent on Solar radiation. In addition, an effective feature extraction scheme was introduced in this work in order to optimize the illuminance prediction.

To sum up, all of the above-mentioned examples of smart street lighting systems across the world mainly focus on energy efficiency, carbon dioxide emission reduction, traffic and population density of an area, and sustainability aspects. However, artificial intelligence (AI) is known to be a potentially powerful tool to enhance the application of current infrastructure [18]. Thus, the focus of our proposed system was on creating a predictive AI model to assist the decision-making process of smart street lighting control on the existing infrastructure with the objective of optimizing energy efficiency while providing sufficient illumination to the campus. This research also contributes to the prediction of illuminance values which is still a scarce topic in weather-forecasting research.

In our previous implementation of the smart street light system installed at Thammasat University, Rangsit Campus, Thailand, Deepaisarn et al. [19] proposed a web application platform for monitoring smart street lighting device status and visualizing data collected by the system, which also connected the flow of data with a city-scale platform of the Thammasat model [20]. The system included 167 smart street lights, which were installed on the campus in February, 2022. Each device was connected to one of the three gateways. In this work, the meteorological and environmental values collected from the environmental sensors were taken into account in order to develop an automatic pipeline to adjust the lighting environment for the campus to an appropriate level. In this regard, the available platform was extended to satisfy greater functionalities and more flexible controls over the devices. By default, the smart street lights along main roads on the campus are scheduled to turn on and off at specified times every day, allowing safety and convenience for the public. That design provides for easy administrative maintenance of the system. However, weather conditions change from time to time due to the influence of the environment, which is uncontrollable by means of a single parameter. Thus, static scheduling does not always guarantee an optimized lighting solution for the campus. Devices may need to turn on or off in response to other weather conditions, which is likely to require shifts from the default scheduled times. It may result in reduced road safety when there is not enough light, or result in excess electrical usage than is necessary. A previous study indicated that the adaptation of a smart street light control system reduced energy consumption up to 25% for industrial and commercial sectors and up to 45% for educational sectors [21]. AI-assisted data analytics was performed in our designed system to enable the prediction of future environmental conditions on the campus using data gathered locally. Models were built to determine the suitable dimming value of smart street light devices and to automatically apply the adjustment on the connected devices accordingly.

The remaining sections of this paper are outlined as follows. Section 2 describes the installed smart street light system infrastructure and hardware requirements of the system. Section 3 explains the functional requirements and process overview of the device control and prediction retrieval functionality, as well as the data used for the creation of the prediction models. Section 4 elaborates on the system API and user interface development of the device control functionality. Section 5 presents the data analytics pipeline to generate prediction models. Section 6 discusses how a prediction model on the data analytics platform is connected to automate the smart street lighting adjustment. Section 7 shows the performance and results of the prediction models. Section 8 discusses the results and the implementation of the work. Finally, the conclusion, the limitations, and the future of this work are summarized in Section 9.

## 2. Infrastructure and Hardware Requirements

**Figure 1:**
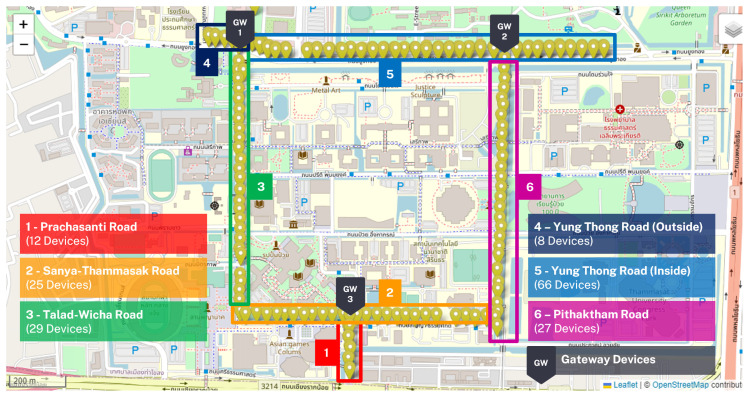
The map of Thammasat University, Rangsit Campus, Thailand, located at 14°04′27″ N, 100°36′08″ E. Yellow location markers display the locations of the 167 smart street lighting devices on the web application. Each color indicates the smart device management zone on the main roads of the campus. Gray markers notated with GW locate the installed location of three gateway devices.

Smart street lighting devices and environmental sensors were installed at Thammasat University, Rangsit Campus, Thailand, at the beginning of 2022. A total of 167 adjustable smart street lights (model ST012-5312-TA(T), MinebeaMitsumi Inc., Tokyo, Japan), equipped with 120-Watt maximum power light-emitting diode (LED) lamps, were installed along the main roads inside the campus. The six smart device management zones were categorized based on the installed location on the main roads of the campus, namely, the Prachasanti, Sanya-Thammasak, Talad-Wicha, Yung Thong, and Pithaktham roads. The map of Thammasat University, Rangsit campus, is illustrated in Figure 1. These devices were installed approximately 20 m from one another to provide a sufficient lighting environment and comply with Thailand’s standard regulation. Each device was also equipped with an illuminance sensor on top of the light pole and a Zhaga node as a control interface. Each Zhaga node device has a maximum power consumption of 0.5 Watts and is capable of Ingress Protection Code of IP66 when installed on street lighting devices. In addition, an environmental station (MinebeaMitsumi Inc., Tokyo, Japan) was installed to observe and collect data, including meteorological and light-related parameters. The sensors can measure illuminance from 0 to 100,000 Lux, temperature from −20 to 50 degrees Celsius, wind speed from 0 to 25 m per second, wind direction from 0.1 to 360 degrees, relative humidity from 0 to 100 percent, and rain level from 0 to 20 mm per hour, etc.

Three gateways are responsible for connecting the devices to the central management suite (CMS), which is an external cloud platform operated by the manufacturer. The gateway devices are located at the intersection of smart device management zones. These devices communicate with other devices in the network by transmitting narrow band radio frequency between 920 to 925 MHz using the IPv6 over Low-Power Wireless Personal Area Networks (6LoWPAN) protocol to create a wireless communication between node devices and gateways with end-to-end device 128-bit AES encryption. The 6LoWPAN allows communication of low bandwidth, low power usage, low cost, and large device deployment over an extensive lifetime.

The external platform, CMS Neptune-SC-v6.0.3 (Paradox Engineering, Novazzano, Switzerland), is operated by the manufacturer of the smart street light devices to provide connectivity and controllability of the devices via an application programming interface (API). The provided API is a representational state transfer (REST) API that can be called upon the user’s request via hypertext transfer (HTTP) protocol. This enabled us to send a customized HTTP request to control devices and retrieve data according to our research and system development requirements.

## 3. Functionalities and Data Requirements

**Figure 2:**
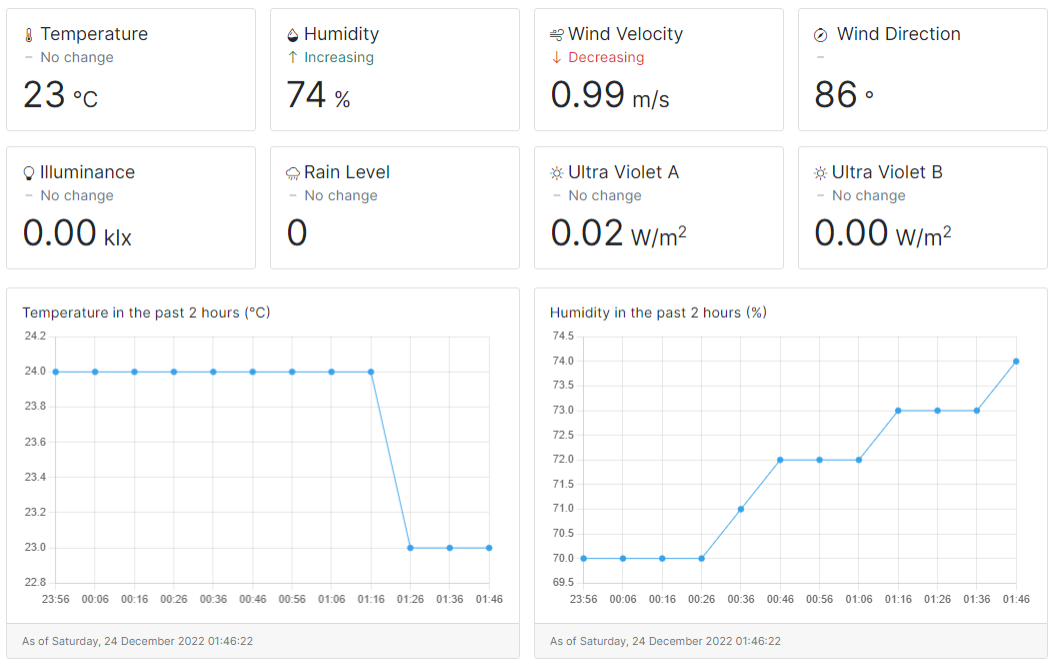
The dashboard shows on the user interface on the web application platform.

According to the proposed web application platform made in our previous work, i.e., Deepaisarn et al. [19], the functionality of the system includes the device connection state monitoring system, environmental data dashboard, and the device disconnection detection system. In the current implementation, the essential data collected using the installed environmental sensor appears on the dashboard, as demonstrated in Figure 2. The dashboard consists of the numerical and graphical format of the data, showing temperature, humidity, wind velocity, wind direction, illuminance, rain level, Ultraviolet A, and Ultraviolet B. These parameters were selected for display on the dashboard due to their roles in the analytical tasks discussed later in this article. Therefore, it was necessary to ensure that the data was collected successfully and correctly.

**Figure 3:**
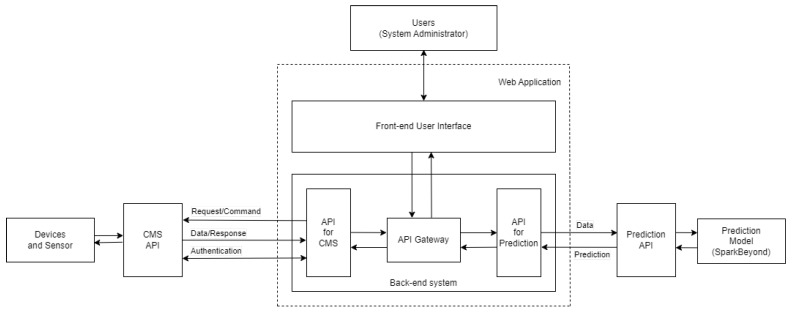
The overview diagram of the process in the smart street lighting web application platform.

In this work, the extensively upgraded version of the platform was implemented with the smart street light manual control system and the automatic light adjusting system that was connected to the prediction model built on the AI-assisted data analytics platform. The manual control system had to be available to control either each device individually or an entire device management zone. The front-end user interface was created to serve system administration and maintenance staff to interact with, and control, the devices. The back-end application programming interface (API), called the hypertext transfer protocol (HTTP), was made once the command was created and confirmed by the user. The back-end API was processed by reading the incoming device control request and its specified dimming value, and then the control command was sent and executed on the CMS API, an external control platform. The process overview diagram is illustrated in Figure 3.

The process for controlling devices by their installed zone was implemented in a similar way to the individual device control. The only difference in implementation was the retrieval of a list of devices in the selected zone by the user, iterating through all devices in the mentioned list, and issuing the same command to the devices. The progress of executing the commands is reported back to the user. Therefore, the API path for sending the control command to an individual device must be created in the back-end system.

After the device control API path is established, the automatic light adjusting system can be implemented with the usage of the prediction model on the separate data analytics platform, called SparkBeyond Discovery Platform (SparkBeyond, Netanya, Israel). The automatic system consists of two parts, the prediction model and the API path to obtain the predicted data. The prediction model created on the data analytics platform has the objective to predict future environmental conditions, based on the data collected from the environmental sensors. Therefore, the collected data must be thoroughly studied and understood. The environmental sensor data are collected every 10 min consecutively all the time. The data can be obtained using the API created by the proposed system in JavaScript Object Notation (JSON) format and later converted into Comma-Separated Value (CSV) format, which is used as inputs to train prediction models. These are time-series data, where each timestamp contains a collection of environmental values, such as temperature, humidity, rain level, and air pressure, and lighting data, such as illuminance, Ultraviolet A, and Ultraviolet B. A detailed explanation of the dataset used in the training process of the prediction model is elaborated on in the next section.

**Figure 4:**
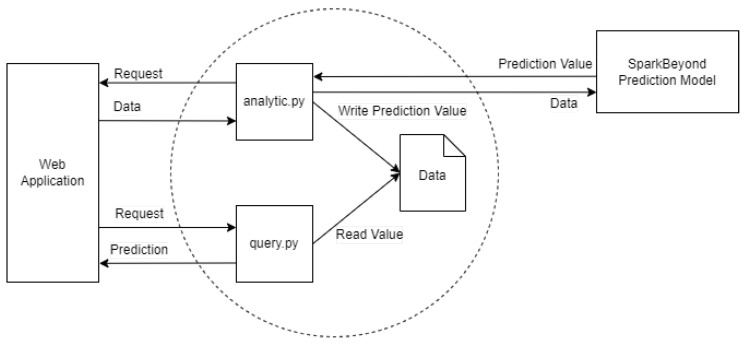
The diagram for processes and components of the prediction API. Sensor data are trained to build a prediction model in the data analytics platform which then returns a suggested dimming value.

The API path for obtaining the predicted data from a prediction model with a suggested light dimming value for smart street light devices had to be created to connect with the current back-end system to build an end-to-end automated smart street light adjustment system. However, Python 3.7 and Flask python web framework [22] were used to develop the API path and obtain the predicted data, in order to be compatible with the data analytics platform used. The application of the flask framework can be seen in various examples, such as the back-end side of the database management system in educational institutions [23]. After that, the prediction API path had to be hosted on the cloud platform and available to use over HTTP protocol. The process of the prediction API is illustrated in Figure 4.

## 4. Device Control

### 4.1. Device Controller API

**Figure 5:**
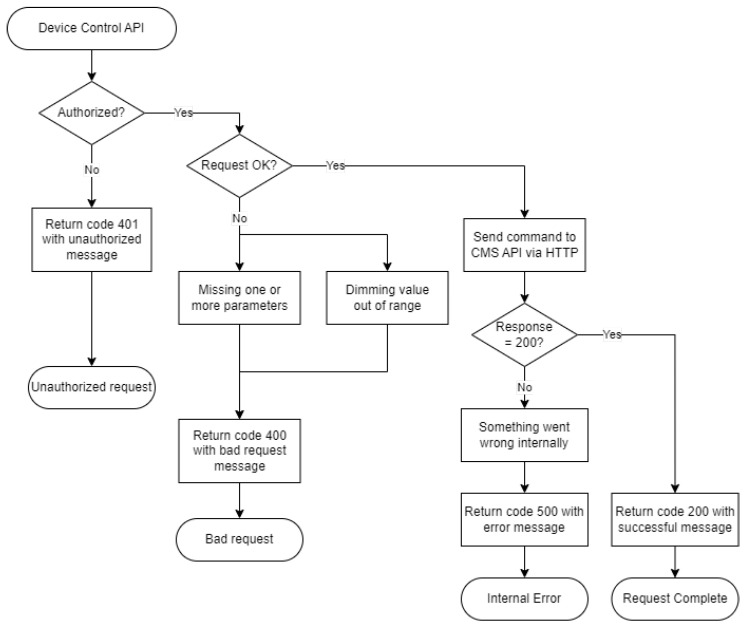
The flowchart shows the API process of controlling individual smart street light devices.

After all requirements of the extended platform were specified, as described in the previous section, the design and development of the API path responsible for controlling an individual device could be performed. The JavaScript language was used to create and develop the API, the language commonly used in many server-side application developments. In particular, Node.js, a JavaScript runtime environment was selected for the development. On top of that, the Express.js [24] framework was also implemented to handle a routing functionality with a higher level of customization to create API paths for this system. However, this back-end system application was a medium between the CMS API and the prediction model API, and, thus, the communication module had to be implemented to allow the systems to communicate with each other. Therefore, the Axios [25] module was used to enable communication over the HTTP protocol. Once the user sends the request with this device control API path, which requires specification of the device identifier and preferred dimming value between 0 to 100, the back-end system first performs the input validation of the request. If one of the required parameters is missing or fails the input validation due to out-of-range dimming value, the HTTP error code 400 and a bad request message are sent back as a response to the user. Otherwise, the process continues with the authentication to the CMS API and the construction of HTTP requests that are sent to the CMS API using the Axios module. Without the authentication to the CMS API process, the back-end system cannot send any request made to any devices in the system. Later, the HTTP request contains the user-specified light-dimming value and designated device identifier that uniquely corresponds to each smart street lighting device. After the HTTP request is sent to the CMS API, the HTTP code 200 and success messages are returned to the user, unless the error occurred during the execution of the command. If the error occurred during that time period, the HTTP error code 500 and message contained with the internal server error and further useful error messages are returned to the user. The flowchart of the individual device control process is shown in Figure 5.

### 4.2. User Interface for Device Control

The user interface for system administrators to control devices, both each individual device and each entire zone, is established on the web application platform to achieve more convenient control and easier access. This interface allows system administrators and maintenance to interact and control the smart street lighting devices, which later interact with an API path created as in the previous section. The interface was developed using JavaScript, which enabled a higher level of customization, such as in handling user events and making HTTP requests. The back-end system development was also developed using JavaScript. Then, the responsive web design was implemented to appropriately fit the web display on different screen sizes without creating all representations of interfaces for various devices and operating systems [26]. Bootstrap CSS framework was applied to this interface because of its mobile-first approach, all browsers support, and, especially, its responsive design [27].

**Figure 6:**
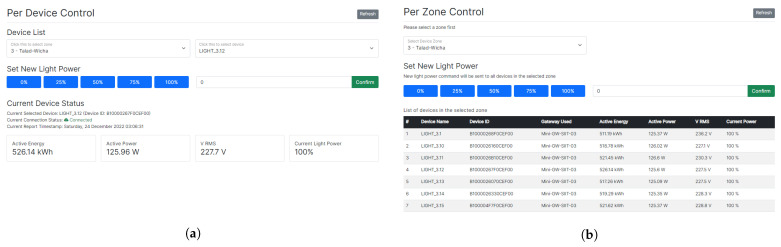
User interface for device control functions. (**a**) Single device control interface. (**b**) Entire zone device control interface.

The individual device control interface contains three critical components: the device selection panel, the light-dimming control panel, and the current operational status of the selected device panel. The device selection panel contains the selection of the device zone and the installed devices, respectively. Once a specific device is selected, the full panels are enabled for users to send their commands, and the current status of the device is shown. These panels are connected to the back-end system developed in the earlier section to provide users with correct functionalities. The current operational status panel shows the current information about the selected device including its current active energy, active power, and light-dimming value. To control the light-dimming value of the device, users can change the value using the light-dimming control panel, which can select from 5 quick-access buttons with predefined values or input the preferred dimming value in the text box. The acceptable range of dimming value in integers ranges from 0% to 100%. After the confirm button is entered, the HTTP POST request to the API path designated for device control is created and sent over the network. The confirmation message is displayed on the interface to communicate with users that the command to control the device was successfully executed. If there is any error during the execution of the command, an error message is alerted to the user instead. The single device control interface is shown in Figure 6a.

The control interface for devices in the entire zone was established with the same critical components but slightly different implementations. The selection of the installed device zone is only displayed on the selection panel. The operational status panel displays current information about all devices in the selected zone as a table. The light-dimming control panel display does not change but the behavior after the command confirmation is different. After users submit the command, the list of devices in the selected zone is obtained and HTTP POST requests that the designated path for device control are created for those devices. The progress of the device control command is reported back to the user. Once all commands are executed, the final successful notification message is shown on display. However, the warning and error message is shown instead if one or more device control commands fail to execute. The user interface for the per-zone device control is shown in Figure 6b.

## 5. Data Analytics and Prediction Models

Methods of performing data analytics and the prediction models for performing experiments in this work are described in this section. First of all, exploratory data analysis was carried out in order to understand the behaviors of the dataset. Then, the main experimental part, utilizing the designed AI-assisted data analytic pipeline, was performed.

### 5.1. Exploratory Data Analysis

**Figure 7:**
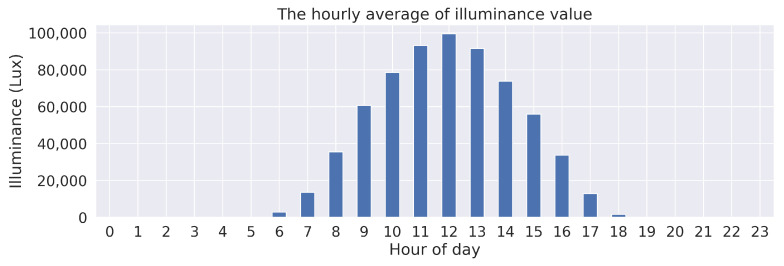
The hourly average of illuminance values over the period of nine months, from February to October 2022.

**Figure 8:**
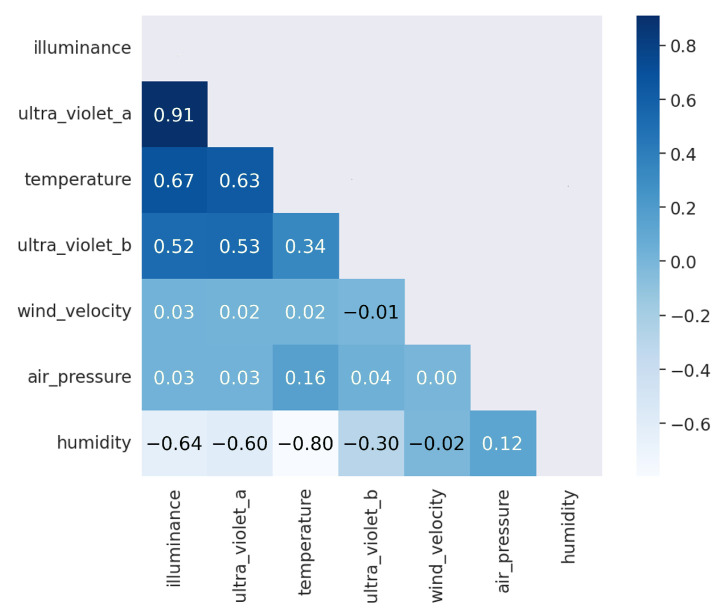
Correlation matrix for the correlation between each pair of variables. The positive strong correlation between Ultraviolet A and illuminance values was investigated.

The purpose of this part is to provide the main data characteristics using statistical graphics and data visualization methods. The first set of analyses examined the hourly average of illuminance value over a period of nine months, from February to October 2022, as illustrated in Figure 7. The natural light illuminance in Thailand seemed to follow a pattern wherein illuminance began to rise around 06:00, peaked in the middle of the day, and then decreased to zero at around 18:00. The correlation matrix, as shown in Figure 8, was investigated to determine the relationship between all features selected from the dataset. According to the correlation matrix, Ultraviolet A and Ultraviolet B had a strong correlation to illuminance values. Thus, these two parameters were removed from the dataset to avoid an over-fitting model. A total of five environmental parameters, including humidity, temperature, air pressure, illuminance, and wind velocity, were preserved as input parameters. The date and time were also included for use as parameters at a training timestamp.

### 5.2. AI-Assisted Data Analytics

Five environmental parameters were included in the data analytics in exploratory data analysis. A data-driven scheme was utilized to empower the management of our smart lighting system by building up models to make better decisions based on intuitive insights. Functionalities in the SparkBeyond Discovery platform (SparkBeyond, Israel) were adopted to provide an AI-driven engine as a tool for extraction of meaningful features and to perform experiments on various machine learning models to learn and solve the time-series problem. The tool has been successfully applied in many research works to gain insight and create efficient models [28,29,30], as well as several industrial applications, ranging from banking, and electronic commerce, to insurance.

#### 5.2.1. Dataset

In this work, the target variable to be predicted by a time-series model was a future natural illuminance value at a specific timestamp. The dataset was collected from the environmental sensors, which were stored in the external platform database and imported via the API [19]. The environmental values from February 2022 to October 2022, were used to train and test the prediction model. The original parameters included humidity, temperature, air pressure, illuminance, wind velocity, and date and time at 10-min intervals. The dataset was cleaned prior to model training.

#### 5.2.2. Experimental Pipeline

First and foremost, hundreds of thousands of features were synthesized from the original parameters in the dataset. The extracted features were then ranked according to their importance, which was assessed using the relative information gain (RIG). Only 50 potential features (out of the 28 million extracted features) were kept for further analyses based on the predetermined feature count that provided significant RIG. After that, the AutoML function available in the SparkBeyond platform [31] was applied so that it generated an optimized predictive model in terms of accuracy and computational costs. The dataset was split into 80% for training and 20% for testing. The next illumination value with a one-day gap was defined as the target in the training phase. In this work, the SciKitLearn standard ML algorithms, consisting of Gradient Boosting, XGBoost, Random Forest, and Decision Tree, were taken into consideration during the model selection process.

### 5.3. Evaluation

For information retrieval, the relative information gain (RIG) was used as the evaluation metric for the feature importance. For model selection, the forecast performance was evaluated by analyzing the characteristics of predicted versus actual illuminance values. Two evaluation metrics were observed. Firstly, the correlation coefficient was calculated to find the relationship between the predicted and the actual values. Secondly, the residual between the predicted and the actual value was computed. Both metrics gave us the trend of the over- and under-prediction characteristics of the models. All metrics are described as follows:

1. Relative Information Gain (RIG): This metric assesses the relative gain of information, given that a particular feature is known. The calculation is based on the information entropy of data and feature and their conditional entropy [32], see Equation (1) [29]. Therefore, the more the feature tells about the data, the better the information gained.
  (1) $$RIG=\frac{H(A)-H(A|B)}{H(A)},$$
  where H(A) is the entropy of the data, *A* and H(A|B) is the conditional entropy of the data *A* given the feature variable *B*. From Equation (1), it is clearly seen that the value for H(A|B) affects the RIG value directly. When the entropy H(A|B) is low, meaning the selected feature introduces a highly certain prediction, the RIG is high, approaching the best possible value of 1.
2. Correlation coefficient (*r*): This metric evaluates linear correlation and returns values between −1 and 1 that indicate the degree to which two variables are related, as expressed in Equation (2). If there is a significant positive correlation between two sets of variables, the coefficient is close to 1. In contrast, if the coefficient is close to −1, the correlation between two sets of variables is strongly negative.
  (2) $$r=\frac{\sum_{i=1}^{m}\left(y_{i}-\bar{y_{i}}\right)\left({\hat{y}}_{i}-\bar{{\hat{y}}_{i}}\right)}{\sqrt{\sum_{i=1}^{m}{\left(y_{i}-\bar{y_{i}}\right)}^{2}\sum_{i=1}^{m}{\left({\hat{y}}_{i}-\bar{{\hat{y}}_{i}}\right)}^{2}}},$$
  where *m* is the number of instances in the test set, *i* is the index at each instance in the test set, possessing a value of 1 to *m*. $y_{i}$ and ${\hat{y}}_{i}$ are the actual and predicted values, respectively. $\bar{y_{i}}$ and $\bar{{\hat{y}}_{i}}$ are the mean of the actual and predicted values, respectively.
3. Residual ($\Delta y_{i}$): This metric computes the difference between the predicted and the actual values at each instant *i*, as expressed in Equation (3).
  (3) $$\Delta y_{i}={\hat{y}}_{i}-y_{i},$$

## 6. Light Adjustment Automation

### 6.1. Prediction Retrieval API

To establish a connection between the back-end system and the prediction model and create a fully-functional automated system, the API for obtaining prediction value was created. As mentioned previously, this API was created using Python 3.7 and Flask web framework which was compatible with the available Software Development Kit (SDK) from the AI-assisted data analytic platform in the SparkBeyond. Two main python scripts were created inside this system to serve the retrieval and connect to the prediction model, called the analytic and the query script. The analytic script was responsible for obtaining the environmental data from the back-end API, sending it to the AI-assisted system for training a prediction model, obtaining the predicted value and its suggested light-dimming level, and writing to the local file in the system. The suggested light-dimming level was determined using the predicted illuminance value by the step function, as shown in Equation (4).

(4) $$f\left(x\right)=\left\{\begin{array}{cc} 100\% & x\leq 300,\\ 75\% & 300<x\leq 600,\\ 50\% & 600<x\leq 900,\\ 25\% & 900<x\leq 1200,\\ 0\% & 1200<x \end{array}\right.$$

where *x* is the predicted illuminance value obtained from the prediction model, and the value for f(x) is the suggested light-dimming level. These threshold values are used in the definition of the step function where the lights are turned on at their maximum power when the illuminance is 300 Lux or lower. This process was executed once a day at midnight to update the existing data in the model. The query script handles the incoming request from the back-end of the web application, queries the predicted value and suggested light-dimming value, and sends it back to the back-end system. This entire API is hosted to run on Azure Virtual Machine, which supports high-performance computing applications [33].

### 6.2. Automated System

With both device control and data prediction API established, the automated street light adjustment system could be initiated. The APIs developed in earlier sections of this paper were used to connect and create the automated system. The automated system ran as a background process in the backend system of the web application. The automated process begins by calling the data prediction API via HTTP to request the predicted illuminance value and suggested light-dimming value, based on the requested timestamp. When these values are returned to the backend system as a response from the prediction API, the device control command is constructed with the suggested light-dimming value obtained. The command is sent to all devices in the system via HTTP using the device control API. After receiving all responses back from the device control API, the process is completed in the case of no error occurring during the command execution process. If one or more errors occur, the automated system sends the same device control command to the failed devices up to three times. Otherwise, the control execution error is logged into the system. However, if any error occurs during the prediction value retrieval process, the automated system retries to obtain the predicted value up to three times. After three unsuccessful retries, the prediction retrieval error is logged into the system. This automation process is executed on a 20-min interval basis, starting from 05:00 to 20:00. The reasoning behind the selected time period was to reduce unnecessary calls to the API during the nighttime when all smart street light devices in the system were expected and set to turn on at 100%.

## 7. Results

The extracted features from the input environmental dataset were ranked by their RIG metric. Table 1 lists the top five features, which are all related to the timestamp and illuminance value. The future timestamp of illuminance was predicted using machine learning algorithms. The performance of each model was evaluated using a correlation coefficient metric to compare the predicted to the actual values. The results in Table 2 indicate the results of the experiment comparing the performance of different machine learning models on the time-series prediction of illuminance values on the environmental dataset. The four selected machine learning models were Gradient Boosting, XGBoost, Random Forest, and Decision Tree with varied analysis window sizes: 3, 4, 5, 6, and 7 days.

In general, a model performs better when it has a higher correlation coefficient between predicted and actual values. The results suggested that the optimal model varied depending on the size of the analysis window. For example, the XGBoost model performed the best among the selected models given a window size of 3 days, yielding the highest correlation coefficient of 0.922. However, at a window size of 7 days, the Random Forest model performed better than others, yielding the highest correlation coefficient of 0.918. Moreover, it appeared that the Decision Tree model consistently had the worst performance, yielding the lowest correlation coefficient for all window sizes. It was possible that the Decision Tree was potentially overfitted, resulting in poor performance in predicting from unseen data.

**Remark** **1.** *Conditions of the original features No. 1–5 used for creating the categorical features of True or False, which is adopted for use as refined features when training machine learning models.*

- *No.1: Let T be the hour of the day,*
$$\delta \left(T\right)=\left\{\begin{array}{cc} True & T\;within\;18:00–05:00\;h,\\ False & otherwise. \end{array}\right.$$
- *No.2: Let* $S_{a}:=\log\left(l_{T}/l_{1}\right)/SD\left(l_{t}\right)$ *, which is Sharpe Ratio,*
$$\delta \left(S_{a}\right)=\left\{\begin{array}{cc} True & S_{a}\in \left(-15.00\times 10^{-4},1.94\times 10^{-4}\right),\\ False & otherwise. \end{array}\right.$$
- *No.3: Let* $CubeRt:=\sqrt[{3}]{l_{T}},$
$$\delta \left(CubeRt\right)=\left\{\begin{array}{cc} True & CubeRt\in (-\infty ,1.956),\\ False & otherwise. \end{array}\right.$$
- *No.4: Let* $L:=l_{T},$
$$\delta \left(L\right)=\left\{\begin{array}{cc} True & L\in (1392,\infty ),\\ False & otherwise. \end{array}\right.$$
- *No.5: Let* $\Delta :=l_{T}-l_{t_{n}},$
$$\delta \left(\Delta \right)=\left\{\begin{array}{cc} True & \Delta \notin (-0.86,469.34),\\ False & otherwise. \end{array}\right.$$

**Figure 9:**
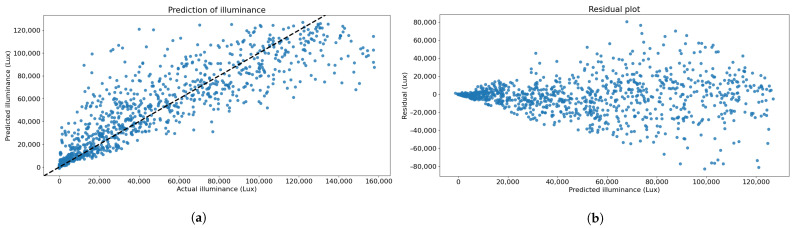
Quantitative evaluation from the experiment using the XGBoost model with a 3-day window size. (**a**) Relationship between predicted and actual illuminance values. (**b**) Plot of the residual against the predicted values.

Figure 9a illustrates the relationship between the predicted and the actual illuminance values of the test datasets where the error trend could be observed. The prediction performance could be investigated using a line graph, ${\hat{y}}_{i}=y_{i}$, where the horizontal and vertical axes represented actual and predicted illuminance values, respectively. The output of an effective prediction model was a predicted value that was close to the actual value and plotted on the linear graph. The residual plot was also important for evaluating the performance of the prediction model. Residuals from a good model should not deviate too far from the horizontal axis. The residual plot from the XGBoost model with a 3-day of window size is shown in Figure 9b. It can be seen that the residual increased with increasing predicted illuminance values. However, the forecast at low illuminance values possessed relatively tiny errors compared to the higher illuminance. The discussion of these results is provided in the next section.

## 8. Discussion

From our implementation of the proposed system in this work, an automated system was established to automatically control smart street lighting devices installed on the campus. The system works based on the predicted illuminance value obtained from the trained prediction model with the AI-assisted data analytic application which suggests the appropriate light-dimming value at a queried instance. While performing the exploratory data analysis, some of the collected parameters from the environmental sensors were excluded from the dataset in the creation of the prediction model to avoid over-fitting behavior. The excluded parameters were Ultraviolet A and Ultraviolet B, due to their physical properties inducing a high correlation with the illuminance value, which was the target value of this prediction model, i.e., visible light and solar ultraviolet radiation were dependent. The terrestrial radiation in the component of Ultraviolet (UV) during midday sun consisted of 95% Ultraviolet A and 5% Ultraviolet B [34]. The finding of a high correlation between Ultraviolet A and illuminance parameters from our exploratory data analysis aligned well with the facts discussed, as shown in Figure 8.

The prediction model using the XGBoost with a 3-day window size was selected due to its highest correlation coefficient between predicted and actual values of 0.922, as shown in Table 2. The results implied that the 50 features extracted and ranked, based on their RIG values, were important and meaningful to predicting illuminance, resulting in a high correlation between the predicted and the actual values. The top five important features shown in Table 1 reflected significant features that led to reliable prediction. The first important feature obviously demonstrated daytime and nighttime effects on illuminance value. The other four remaining important features were also directly related to illuminance value, such as the increasing or decreasing trends of the value, which were refined into the final set of features used for building the prediction model, see the Remarks in Table 1. The model accurately predicted illuminance values for the next day’s offset, given the periodic patterns learned from the previous three days of data. It was found that the hour of the day played a significant role in determining natural light illuminance. A high-performance prediction model could be achieved, providing these timestamp-based features. This study did not find a significant difference when varying the window size of data used for training, as stated in Table 2. There are several factors why increasing the training period did not lead to any improvement in prediction performance. Firstly, the model had already learned the daily patterns from the three days of data. The additional training window size, taking into consideration a period longer than three days, might not add much meaningful information to the model. Secondly, a longer period of training data could potentially introduce additional variations in the data that could affect the model’s ability to learn and make accurate predictions, requiring more complexity or fine-tuning techniques for model optimization. These findings were in agreement with the finding of Ferrari et al., which suggested that the illuminance had a daily seasonality, i.e., a 24-h periodic pattern. Moreover, the more model variables included in computation, the greater errors of value estimation and time taken for training the model were observed [35]. While the highest correlation coefficient was obtained by the selected model parameters, the plot of the residual of actual and predicted values showed that the residual increased with the value of illuminance. Despite this, the residual at the higher end of the predicted illuminance values was not essentially considered for the analysis in this study. In other words, the only important period of prediction for the automated light-adjusting process was during the day-night transition. Therefore, the errors observed in Figure 9b resulted in reliable natural illuminance prediction which allowed reasonable adjustment of light-dimming stages.

## 9. Conclusions, Limitations, and Future Work

This work developed and implemented an automated light-adjusting system, based on the suggested value from a prediction model created using an AI-assisted data analytic pipeline for the smart street lighting system, installed at Thammasat University, Rangsit Campus, Thailand. The proposed system consists of the device control API, which is connected to the external control platform, and the prediction retrieval API, which is connected to the AI-assisted platform. The device control API establishes the user interface for system administrators to manually control devices by individual device or by zone. The prediction model was created using the XGBoost machine learning model and three-day window size settings, which provided the best-performance model in this work with a correlation coefficient of 0.922. The automated system connected the APIs and executed them every 20 min. The step function was developed to suggest the light-dimming level adaptively, according to the predicted natural illuminance at an instance to ensure the visibility of drivers and the safety of pedestrians in the surrounding area. This current proof-of-concept for the automatic and optimal process was implemented on the real infrastructure.

One of the major concerns of the current system implementation was the population density and movement of pedestrians in the surrounding area, which in this work was not taken into consideration. In the future, this motion detection subsystem could be implemented as an extension and further improve the system. Moreover, further work on the optimization of the web application platform and the connection between its underlying subsystem will continue to improve. The validation and user acceptance testing of the current user interface and its user experience could be done with a group of system administrators and maintenance staff in the near future to ensure the web application is more user-friendly. Furthermore, this system with the integration of an AI-assisted platform could be implemented in other campuses/cities and other sectors, such as agriculture, through their own data collection solution and infrastructure requirements. This smart street lighting system enhances energy efficiency and promotes sustainability on the campus, according to the sustainable development agenda of Thammasat University and the sustainable development goals of the United Nations.

## Figures and Tables

**Table 1:** The top five important features according to the relative information gain (RIG) score: The window size for the timestamp was determined by the training period, which was three days for the input and one day for the offset, and total data for a 4-day period. The illuminance value in this period could be listed in the set, $l_{t}:=\left\{l_{t_{1}},l_{t_{2}},l_{t_{3}},\ldots l_{t_{n}},l_{T}\right\}$, where *T* was the last timestamp in the training phase and SD was the standard deviation.

No.	Feature	Direction of Effect	Train RIG
1.	*T*	Lower illuminance	0.416
2.	$\log\left(l_{T}/l_{1}\right)/SD\left(l_{t}\right)$	Higher illuminance	0.394
3.	$\sqrt[{3}]{l_{T}}$	Lower illuminance	0.375
4.	$l_{T}$	Higher illuminance	0.366
5.	$l_{T}-l_{t_{n}}$	Lower illuminance	0.365

**Table 2:** Correlation coefficient of predicted and actual illuminance values assessed on the test data using the models trained with varying machine learning model and window size.

	Window Size	3 Days	4 Days	5 Days	6 Days	7 Days
Model						
Gradient Boosting		0.918	0.914	0.919	0.912	0.912
XGBoost		0.922	0.920	0.920	0.919	0.903
Random Forest		0.919	0.917	0.918	0.915	0.918
Decision Tree		0.839	0.840	0.848	0.838	0.840

## Data Availability

The dataset collected from the environmental sensor installed at Thammasat University, Rangsit Campus, Thailand, during the development of this paper is available to download at the following link https://siit-smart-city.azurewebsites.net/csv-download (accessed on 9 November 2022).

## Abbreviations

The following abbreviations are used in this manuscript:

6LoWPAN	IPv6 over Low-Power Wireless Personal Area Networks
AI	Artificial Intelligence
API	Application Program Interface
CMS	Central Management Suite/System (MinebeaMitsumi Inc., Japan)
CSS	Cascading Style Sheets
CSV	Comma-Separated Value
CO_2_	Carbon Dioxide
HPS	High Pressure Sodium
HTTP	Hypertext Transfer Protocol
LED	Light-emitting Diode
ML	Machine Learning
IoT	Internet of Things
JSON	JavaScript Object Notation
RCMS	Remote Control and Monitoring System
REST	Representational State Transfer
RIG	Relative Information Gain
SD	Standard Deviation
SDK	Software Development Kit
